# A new suite of *tnaA* mutants suggests that *Escherichia coli* tryptophanase is regulated by intracellular sequestration and by occlusion of its active site

**DOI:** 10.1186/s12866-015-0346-3

**Published:** 2015-02-04

**Authors:** Gang Li, Kevin D Young

**Affiliations:** Department of Microbiology and Immunology, University of Arkansas for Medical Sciences, Little Rock, AR 72205-7199 USA

**Keywords:** TnaA, tryptophanase, Cell pole, Protein localization, Enzyme regulation

## Abstract

**Background:**

The *Escherichia coli* enzyme tryptophanase (TnaA) converts tryptophan to indole, which triggers physiological changes and regulates interactions between bacteria and their mammalian hosts. Tryptophanase production is induced by external tryptophan, but the activity of TnaA is also regulated by other, more poorly understood mechanisms. For example, the enzyme accumulates as a spherical inclusion (focus) at midcell or at one pole, but how or why this localization occurs is unknown.

**Results:**

TnaA activity is low when the protein forms foci during mid-logarithmic growth but its activity increases as the protein becomes more diffuse, suggesting that foci may represent clusters of inactive (or less active) enzyme. To determine what protein characteristics might mediate these localization effects, we constructed 42 TnaA variants: 6 truncated forms and 36 missense mutants in which different combinations of 83 surface-exposed residues were converted to alanine. A truncated TnaA protein containing only domains D1 and D3 (D1D3) localized to the pole. Mutations affecting the D1D3-to-D1D3 interface did not affect polar localization of D1D3 but did delay assembly of wild type TnaA foci. In contrast, alterations to the D1D3-to-D2 domain interface produced diffuse localization of the D1D3 variant but did not affect the wild type protein. Altering several surface-exposed residues decreased TnaA activity, implying that tetramer assembly may depend on interactions involving these sites. Interestingly, changing any of three amino acids at the base of a loop near the catalytic pocket decreased TnaA activity and caused it to form elongated ovoid foci in vivo, indicating that the alterations affect focus formation and may regulate how frequently tryptophan reaches the active site.

**Conclusions:**

The results suggest that TnaA activity is regulated by subcellular localization and by a loop-associated occlusion of its active site. Equally important, these new TnaA variants are immediately available to the research community and should be useful for investigating how tryptophanase is localized and assembled, how substrate accesses its active site, the functional role of acetylation, and other structural and functional questions.

**Electronic supplementary material:**

The online version of this article (doi:10.1186/s12866-015-0346-3) contains supplementary material, which is available to authorized users.

## Background

The enzyme tryptophanase hydrolyzes tryptophan to form indole, pyruvate and ammonia, and is produced by over 85 gram-positive and gram-negative bacteria [1,2]. The indole thus produced diffuses across cell membranes independent of specific transporters and affects the physiology of nearby members of the microbial community or their animal hosts [2-6]. For example, indole regulates such diverse microbial processes as motility, biofilm formation, antibiotic resistance, persister formation and host cell invasion [2]. Recent observations continue to underscore the biological significance of this compound, including the fact that some bacteria which do not themselves produce indole nevertheless detect its presence and become more antibiotic resistant [7,8], and in others indole inhibits quorum sensing and growth [9]. Finally, *in situ* bacterial production of indole helps create and maintain proper epithelial cell function in the mammalian intestine [10,11]. This broad range of intra- and inter-kingdom effects makes it important to understand more fully how indole synthesis is regulated.

In *Escherichia coli*, tryptophanase is encoded by the *tnaA* gene in the *tnaCAB* operon, which also includes the tryptophan-specific transporter TnaB [12-14]. Transcription of *tnaAB* is activated by the cAMP-CRP complex [15], and is induced by tryptophan via a Rho-dependent terminator and a TnaC leader peptide [16,17]. Induction of the *tnaA* gene and production of indole requires high concentrations of tryptophan, achieved mainly by the import of exogenous tryptophan via the TnaB transporter [18,19]. Functional TnaA is tetrameric, with a pyridoxal phosphate (PLP) coenzyme covalently linked to the Lys270 residue at each of the four active sites [20,21]. Incubation of the enzyme at low temperature results in reversible loss of PLP, with the tetramer disassociating into inactive dimers [22]. Two crystal structures of apo TnaA have been solved; both are tetramers in which each of the four TnaA proteins has a similar quaternary structure comprised of three subdomains: D1, D2 and D3 [23,24]. In turn, subdomains D1 and D3 form a small domain, with D2 forming a single large domain. Interactions between these two domains and with the other three TnaA subunits create the tightly assembled tetramer. The two extant TnaA crystal structures differ in that the catalytic pocket of one is closed [23] while that of the other is open [24]. The existence of these two forms suggests that the structure of TnaA is flexible, though the causes and consequences of this difference are unknown.

Recently, we found that wild type TnaA co-purifies with membrane vesicles derived from the cell poles of *E. coli* and that TnaA-sfGFP forms a single focus at midcell or at one of the poles during mid-log growth but becomes diffusely localized as the cells approach stationary phase [25]. Untagged TnaA competes with TnaA-sfGFP for polar localization, the foci do not have the characteristics of inclusion bodies, and TnaA-sfGFP is as active as the untagged protein with regard to indole production, all of which suggest that TnaA naturally localizes to the pole [25]. However, the mechanism that drives the formation of these foci is unknown, as is the question of whether they have a biological function.

Here, we show that indole production is associated with the disappearance of discrete TnaA foci and the concomitant rise of diffusely localized cytoplasmic TnaA. In tandem, we created six truncated variants of TnaA and 36 missense mutants in which different combinations of 83 surface-exposed polar amino acid residues were converted to alanine. Several variants displayed different localization characteristics and/or reduced enzymatic activities, suggesting that the sequestration and release of TnaA from intracellular foci may represent a novel post-translational mechanism to regulate tryptophanase activity. Finally, three mutations at the base of an external loop may alter TnaA activity by regulating the frequency with which this loop occludes the enzyme’s active site.

## Results

### Diffuse TnaA protein is functional

**Figure 1:**
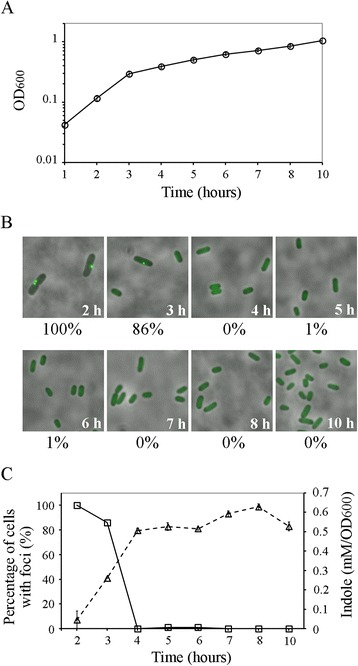
**Indole is produced when TnaA becomes diffuse.** *E. coli* cells (GL619) expressing TnaA-sfGFP from the constitutive *tufA* promoter were grown in M9 minimal medium supplemented with 1% casamino acids and 0.5 mM tryptophan. **A**. Cell growth (OD_600_). **B**. TnaA-sfGFP localization at different times. GFP fluorescence and phase contrast images are overlaid. The percentage of the population that has a polar or midcell focus is indicated below each panel; 50–100 cells were examined for each sample. **C**. TnaA-sfGFP focus formation (squares) plotted against amount of indole produced (triangles). The percentage of cells with TnaA-sfGFP foci are plotted from the data in B. Three independent cultures were assayed for indole production, and the mean ± standard deviation is presented.

TnaA forms a single polar or midcell focus in all cells during mid-logarithmic growth, but the protein gradually diffuses into the cytoplasm as the culture nears stationary phase so that the majority of cells do not contain TnaA foci [25]. Interestingly, indole production is low during early exponential growth but increases dramatically as TnaA becomes more diffuse [25,26]. Under these same conditions, TnaA forms polar foci in 100% of the cells during exponential growth, but when these cells enter stationary phase TnaA diffuses so that only 31-58% of cells contain individual foci [25]. Thus, indole production may increase because more enzyme is made or because TnaA is moved from intracellular foci to become soluble and active in the cytoplasm. In these previous experiments, *E. coli* was grown in LB medium and transited rapidly from mid-logarithmic growth into stationary phase, so it was difficult to determine if the diffuse TnaA fraction was responsible for indole production. Therefore, to address this relationship, we incubated *E. coli* in M9 minimal medium to slow cell growth. We also examined TnaA function in *E. coli* GL619, a strain in which the chromosomal *tnaA-sfgfp* gene is controlled by a *tufA* promoter. In this strain, the TnaA-sfGFP fusion protein is produced steadily and constitutively [19], allowing us to observe the regulation of TnaA activity in the absence of any effects that might be caused by alterations in *tnaA* gene expression. As expected, TnaA-sfGFP formed single polar or mid-cell foci during early mid-logarithmic growth (Figure 1A and B, 2 h) but became completely diffuse as the cells entered stationary phase and thereafter (Figure 1B, 4 h and afterwards). Most interestingly, indole was not produced during mid-logarithmic growth (Figure 1C, 2 h) but was produced continuously after TnaA had become diffuse in stationary phase (Figure 1C, 4 h and afterwards). These results suggested that indole was produced by the diffuse TnaA fraction but was not produced by TnaA sequestered in a focus.

TnaA probably dimerizes before assembling into the functional tetramer. Thus, the diffuse and focus fractions might be composed of different multimeric forms of TnaA, and, if so, the formation of TnaA foci might regulate TnaA function. Unfortunately, repeated attempts to isolate and analyze intact foci were unsuccessful because the foci dissolved and disappeared when the cells were disrupted. This prevented us from analyzing the multimerization status of TnaA in foci and also prevented us from determining the precise amount of soluble TnaA in cells as they progressed through the growth cycle. However, because these lysates did convert tryptophan to indole (not shown), the results indicated that foci were easily disrupted to give fully active TnaA-sfGFP tetramers.

### The TnaA D1D3 two domain dimer localizes to cell poles

**Figure 2:**
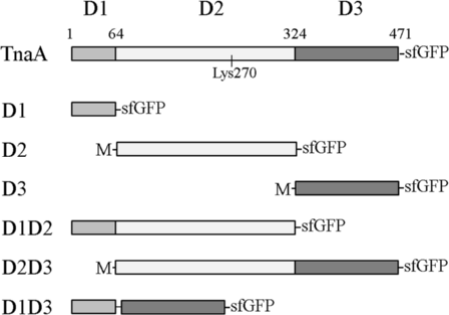
**Construction of TnaA truncation mutants.** The regions of the D1, D2 and D3 subdomains were determined according to the TnaA crystal structure (PDB 2OQX). The position of the active site Lys270 is indicated. In D2, D3 and D2D3, the starting methionine codon was retained. In D1D3, a short linker (Ala-Ala-Gly-Tyr-Asp) was inserted between the two subdomains. All proteins were constructed to contain a C-terminal sfGFP. Each gene was inserted into the chromosome to replace the wild type *tnaA* gene at that position.

**Figure 3:**
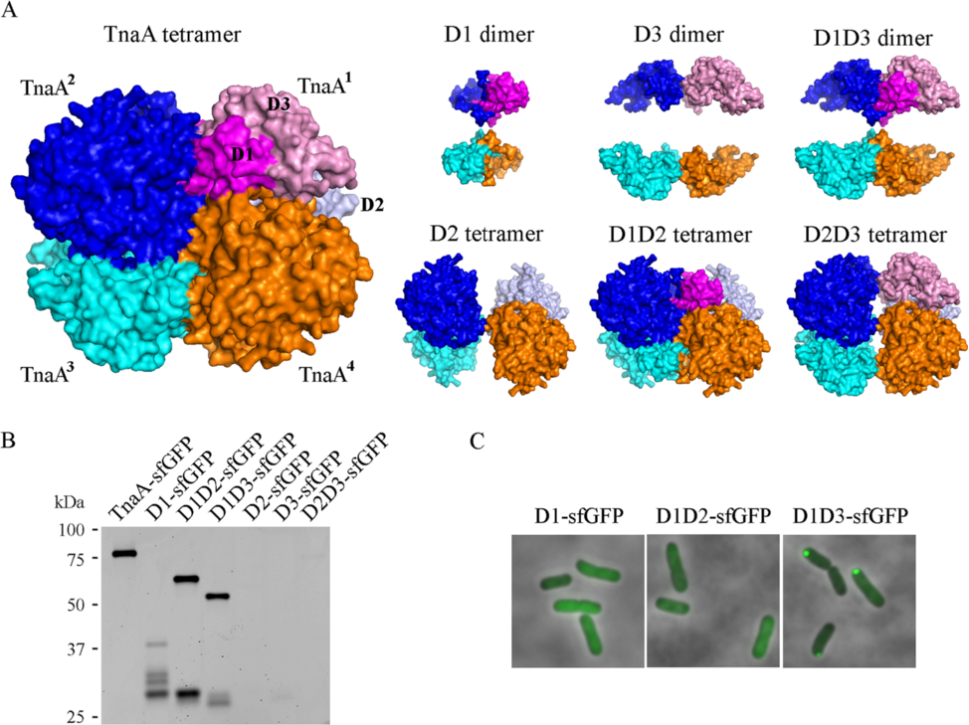
**The D1D3 domain is sufficient for polar localization. A**. Models of the oligomerization status of TnaA and the subdomain constructs. The four subunits in the TnaA tetramer are designated as TnaA^1^, TnaA^2^, TnaA^3^ and TnaA^4^. The deleted subdomains were removed from the TnaA tetrameric crystal structure (PDB 2OQX). The positions of the D1 (magenta), D2 (grey) and D3 (pink) subdomains in TnaA are illustrated in the upper right molecule of the tetramer. The other three TnaA molecules that make up the tetramer are shown in blue, cyan and orange. The four catalytic pockets are located at the left and right sides of the tetramer, and are not visible in this view. **B**. Protein stability of the subdomain constructs. *E. coli* strains expressing each TnaA truncation mutant were grown in LB to an OD_600_ of 1.0 and analyzed by SDS-PAGE. The sfGFP fusion proteins were visualized by in-gel GFP fluorescence imaging. The top-most band in each lane represents the expected size of the respective full-length fusion protein. **C**. Cellular localization of the subdomain constructs. GFP and phase contrast images are overlaid.

Because the TnaA in discrete foci could not be tested separately from the diffuse protein, we used a truncation and mutagenesis approach to address the question of how TnaA multimerization might affect enzyme localization and activity. We rearranged the *tnaA* gene to create a set of derivatives that encoded all six possible combinations of the D1, D2 and D3 protein subdomains (Figure 2). The *sfgfp* gene was appended to the 3′ end of each rearranged gene, and the fusions were inserted into the chromosome to replace the wild type *tnaA* gene. The resulting gene constructs were expressed under control of the native *tnaA* promoter and Shine-Dalgarno sequence. We modeled the structures of these TnaA variants by subtracting individual subdomains from the known crystal structure (Figure 3A). The truncations predicted the existence of three potential dimers (D1-D1, D3-D3 and D1D3-D1D3) (Figure 3A, upper right three structures) and three tetramers (created by self-association of the D2, D1D2 or D2D3 domains) (Figure 3A, lower right three structures). Note that the tetramer form of TnaA required the presence of the D2 domain, but that different dimers could assemble in its absence.

Cells expressing the D1, D1D2 and D1D3 proteins produced fluorescent products (Figure 3C), but little or no fluorescent signal was observed in cells carrying the D2, D3 and D2D3 fusion products (not shown), suggesting that these latter proteins were not expressed or were degraded. SDS-PAGE analysis of the fusion proteins was consistent with these results (Figure 3B). Among the constructs, D1D3 was the most stable (e.g., exhibited the least degradation), D1D2 was of intermediate stability, while most of the D1 domain construct was degraded into smaller fragments (Figure 3B). The D2, D3 and D2D3 subdomain variants were not visible on SDS-PAGE, strongly suggesting that the D1 domain was essential for the folding or stability of TnaA. None of the truncation variants could produce indole (not shown), confirming that the complete TnaA protein was required for enzyme activity. The D1D3-sfGFP fusion protein formed a single focus at midcell or at one of the poles, as was observed for full length TnaA-sfGFP (Figure 3C). In contrast, the D1 and the D1D2 variants were diffuse throughout the cytoplasm (Figure 3C). Thus, the D1D3 dimer was sufficient for directing polar localization. Because the D1-only and D3-only variants were not stable, we could not determine directly if one or the other subdomain contained the major localization signal.

### The polar localizations of TnaA and D1D3 are not artifactual

**Figure 4:**
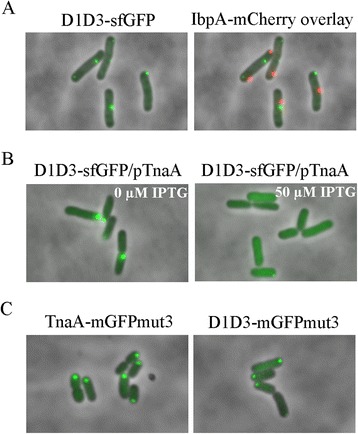
**The polar localizations of TnaA and D1D3 are not artifactual. A**. D1D3-sfGFP foci do not overlap with inclusion bodies in *E. coli* GL730 cells coexpressing D1D3-sfGFP and IbpA-mCherry, which binds to protein inclusion bodies. The sfGFP image (left) and the mCherry overlay image (right) are shown. **B**. Plasmid-borne untagged TnaA (from plasmid pTnaA) competes with chromosomal D1D3-sfGFP for polar localization. D1D3-sfGFP (in strain GL726) was localized at cell poles when TnaA was not induced (left), but became diffuse when TnaA was induced (right). **C**. TnaA-mGFPmut3 (left) and D1D3-mGFPmut3 (right) form single foci at the cell poles in strains GL676 and GL734, respectively.

TnaA-sfGFP appears to localize specifically to cell poles because the protein does not co-localize with IbpA, which binds to inclusion bodies, and because TnaA-sfGFP foci are chased from the poles by untagged TnaA [25]. In addition, the dissipation of TnaA foci when the cells were broken open (see above) is unlike the behavior of inclusion bodies, which cohere and can be pelleted by low speed centrifugation. Nonetheless, to determine if the D1D3-sfGFP foci were inclusion bodies, the fusion protein was expressed in the presence of IbpA-mCherry, which binds to misfolded proteins and marks such artifacts [27,28]. Very little IbpA-mCherry signal was observed (not shown), indicating that few misfolded proteins were present to induce *ibpA*. In addition, when the fluorescence images were artificially enhanced, any IbpA-mCherry foci that were present did not overlap with the D1D3-sfGFP foci, confirming that the latter were not non-specific inclusion bodies (Figure 4A). In addition, we overproduced untagged wild type TnaA (from plasmid pTnaA) and found that this protein displaced polar D1D3-sfGFP foci so that the fusion protein was distributed diffusely throughout the cytoplasm (Figure 4B). These results strongly suggest that, like wild type TnaA, the localization of D1D3-sfGFP at the poles was real and specific.

Recent observations indicate that some fluorescent reporter proteins, including sfGFP, may skew the *in vivo* localization behavior of proteins on a case-by-case basis [29]. For example, sfGFP-tagged ClpP forms a single focus but untagged ClpP and a ClpP-mGFPmut3 fusion protein are instead spread diffusely throughout the cytoplasm [29]. Thus, to be doubly certain that polar localization of TnaA was not an artifact caused by attaching sfGFP, we appended monomeric mGFPmut3 to the carboxyl termini of TnaA and D1D3. As before, the fusion proteins localized to the cell poles (Figure 4C), providing further evidence that the polar localization of TnaA was not an artifact caused by adding the sfGFP reporter. In subsequent localization experiments, we observed the behavior of sfGFP-fused proteins because mGFPmut3 produced a much weaker fluorescent signal.

### Residues that affect assembly and localization of TnaA foci

**Figure 5:**
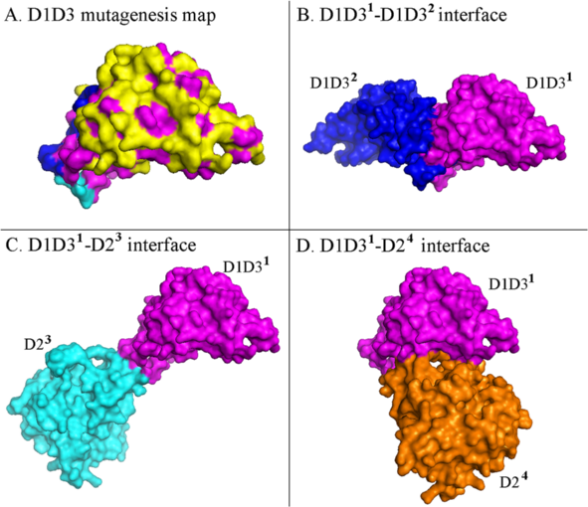
**D1D3 surface residues that affect polar localization of TnaA-sfGFP. A**. Magenta: residues that were not mutagenized. Yellow: alanine substitution mutations that did not affect polar localization of D1D3 or TnaA. Deep blue: mutations that delayed assembly of TnaA foci (mutation sets A2-TnaA, A24-TnaA and A25-TnaA). Cyan: mutations that produced diffuse localization of the D1D3-sfGFP TnaA construct (A9-D1D3). Mutations that produced ovoid foci are not visible in this view and are illustrated in Figure 7. The interfaces between D1D3^1^ and other subunits are illustrated in **B** to **D**. Note that mutations causing delayed focus disassembly at stationary phase (Table 3) are not illustrated.

To identify the functional roles of the regions and residues of TnaA, we mutated 79 charged and polar residues on the surface of the D1D3 construct, reasoning that one or more of these residues might form ionic or hydrogen bonds with neighboring TnaA subunits or with other molecules. These residues covered almost the entire surface of the D1D3 protein (Figure 5A, residues colored yellow, blue and cyan). Non-polar residues were not mutated, although they might contribute additional interactions (Figure 5A, residues in magenta). In addition, six lysine residues (Lys5, Lys450, Lys459 on the D1D3 domains; and Lys115, Lys156, Lys239 on the D2 domain) are known to be acetylated, though the physiological functions of this modification are not known [30,31]. Therefore, in addition to the lysine residues already changed on D1D3, we also mutated the three lysine residues on the D2 domain. Finally, we mutated the active site Lys270 on the D2 domain to determine if PLP or substrate binding might affect protein localization. In sum, 83 different residues were replaced with alanine. As a preliminary screen, we constructed TnaA variants containing one to four mutations near enough to one another that they could be altered by using a single oligonucleotide during site-directed mutagenesis (Tables 1, 2 and 3, mutants A1-TnaA to A32-TnaA). A corresponding series of mutagenized variants was engineered into the D1D3 fusion protein (Tables 1, 2 and 3, A1-D1D3 to A32-D1D3). sfGFP was fused to the carboxyl terminus of each TnaA or D1D3 variant, and the gene for each mutant was inserted into the original chromosomal position so that gene expression was controlled by the native *tnaA* promoter.

**Table 1:** **Mutations that do not affect TnaA localization or enzyme activity (Category 1)**

**Variant**	**Residue(s) changed to alanine**	**Residue location** ^**a**^	**TnaA-sfGFP localization** ^**b**^	**D1D3-sfGFP Localization** ^**c**^	**Indole** ^**d**^ **(%)**
A1	K5	D1 surface	Wild type	N/A^e^	96.5
A3	E17, K20, R21	D1 surface	Wild type	Wild type	101.3
A4	T23, R24, Y26	D1 surface	Wild type	Wild type	99.5
A6	K33, S34	D1 surface	Wild type	Wild type	102.7
A10	K115	D2 surface	Wild type	N/A	93.0
A11	K156	D2 surface	Wild type	N/A	98.7
A12	K239	D2 surface	Wild type	N/A	100.1
A14	N327, D329	D3 surface	Wild type	Wild type	96.8
A15	Q339, Y340, D343	D3 surface	Wild type	Wild type	99.6
A16	E346, E347	D3 surface	Wild type	Wild type	93.1
A18	D363, K366	D3 surface	Wild type	Wild type	97.7
A19	H370, D374, Q375	D3 surface	Wild type	Wild type	99.2
A21b	R403	D3 loop	Wild type	N/A	103.3
A21c	D404	D3 loop	Wild type	N/A	105.4
A22	K406, K409, Q410	D3 loop	Wild type	Wild type	100.9
A26	E437, K440, H441	D3 surface	Wild type	Wild type	92.7
A27	K443, E444, N445, N448	D3 surface	Wild type	Wild type	103.4
A28	K450	D3 surface	Wild type	N/A	97.9
A30	K459	D3 surface	Wild type	N/A	102.9
A31c	T465	D3 surface	Wild type	N/A	72.7
A32	K467, K469, E470	D3 surface	Wild type	Wild type	92.4
A33	K5, K115, K156, K239, K450, K459	Surface	Wild type	Wild type	82.5

^a^Residue location is based on the crystal structure of TnaA (PDB ID 2OQX). Pocket, the active site cavity; loop, the loop comprised of aa 398 to 416 at the edge of the catalytic pocket.

^b^Wild type: spherical focus at midcell or one of the poles at mid-to-late exponential phase, becoming more diffuse at stationary phase as wild type TnaA.

^c^Wild type: spherical focus at midcell or one of the poles at mid-to-late exponential phase. D1D3 constructs were mostly degraded at stationary phase.

^d^The amount of indole produced by each TnaA-sfGFP variant compared to that produced by wild type TnaA in LB at an OD_600_ of 1.5. Values are reported as the percentage of wild type, and are the average of two independent cultures. Growth rates of all cells carrying wild type TnaA or any of its variants were nearly identical.

^e^N/A indicates that the variant was not constructed or that data was not available.

**Table 2:** **Mutations that alter TnaA focus formation during exponential growth (Category 2)**

**Variant**	**Residue(s) changed to alanine**	**Residue location** ^**a**^	**TnaA-sfGFP localization** ^**b**^	**D1D3-sfGFP Localization** ^**c**^	**Indole** ^**d**^ **(%)**
A2	E9, R12, R14	D1D3^1^-D1D3^2^ interface	Delayed	Wild type	7.2
A24	T426, Y427, T428	D1D3^1^-D1D3^2^ interface	Delayed	Wild type	8.5
A25	Q429, T430, H431, D433	D1D3^1^-D1D3^2^ interface	Delayed	Wild type	13.3
A9	T60, Q61, S62, Q64	D1D3^1^-D2^3^	Wild type	Diffuse	5.3
A21	S398, R403, D404	Edge of the pocket	Ovoid	Wild type	5.8
A21a	S398	Bottom of the loop	Ovoid	Wild type	43.4
A23	E416, R419	Edge of the pocket	Ovoid	Wild type	5.1
A23a	E416	Bottom of the loop	Ovoid	N/A^e^	12.1
A31	R462, H463, T465	Edge of the pocket	Ovoid	Wild type	2.1
A31a	R462	Bottom of the loop	Ovoid	N/A	57.1

Legend is the same as for Table 1.

**Table 3:** **Mutations that delay TnaA focus disassembly in stationary phase (Category 3)**

**Variant**	**Residue(s) changed to alanine**	**Residue location** ^**a**^	**TnaA-sfGFP localization** ^**b**^	**D1D3-sfGFP localization** ^**c**^	**Indole** ^**d**^ **(%)**
A2	E9, R12, R14	D1D3^1^-D1D3^2^ interface	Delayed	Wild type	7.2
A5	R27, E28, E29	D1 surface	Wild type	Wild type	14.9
A7	D42, S43, E44, D45	D1 surface	Wild type	Wild type	36.8
A8	D49, T52, D53, S54	D1D3^1^-D2^4^	Wild type	Wild type	6.6
A9	T60, Q61, S62, Q64	D1D3^1^-D2^3^	Wild type	Diffuse	5.3
A13	K270	D2 active site	Wild type	N/A	10.9
A17	C352, Q353, Q354	D3 pocket	Wild type	Wild type	6.0
A20	E384, K387, R392	D3 surface (EK)	Wild type	Wild type	4.8
		D1D3^1^-D2^4^ (R)			
A21	S398, R403, D404	Edge of the pocket	Ovoid	Wild type	5.8
A21a	S398	Bottom of the loop	Ovoid	Wild type	43.4
A29	T453, T455, Y456, E457	D3 surface	Wild type	Wild type	11.7

Legend is the same as for Table 1.

The localization and enzymatic phenotypes of the mutant proteins fell into three categories. In the first category, the mutant proteins behaved like wild type TnaA (Table 1). Each of these variants formed a single polar or midcell focus during the mid-to-late growth phase, after which the proteins became diffuse as the cells entered stationary phase. In addition, these proteins produced indole at wild type levels, indicating that they had normal enzymatic activity. Of particular note is that neither localization nor enzyme activity was affected by mutating individual lysine residues that would normally be acetylated (Table 1, mutants A1, A10, A11, A12, A28 and A30). In fact, a mutant in which all six acetylated lysine residues were mutated continued to exhibit wild type localization and its enzymatic activity was affected very little (Table 1, mutant A33). Thus, many surface residues did not contribute significantly to TnaA function, and the purpose of lysine acetylation remains unknown.

**Figure 6:**
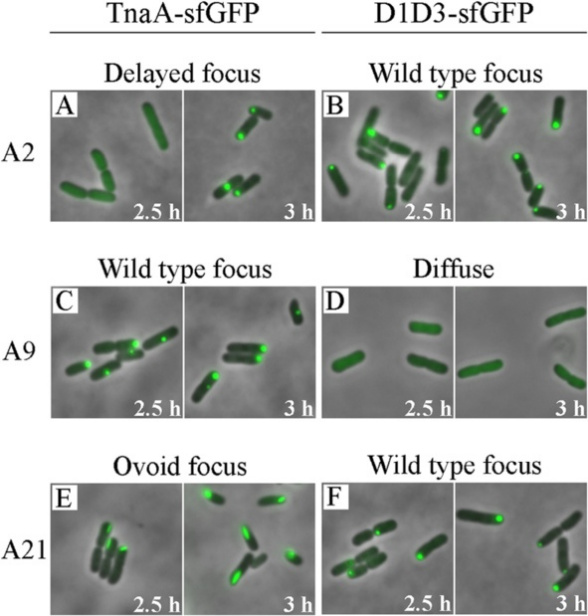
**Altered localization of TnaA and D1D3 variants.** Images of altered localization, one representative variant from each subgroup in Table 2. The TnaA variants are shown in **A**, **C** and **E**, and their corresponding D1D3 variants are shown in **B**, **D** and **F**. The cells were grown in LB for 2.5 or 3 h (OD_600_ ~ 0.6 and 1.0, respectively).

**Figure 7:**
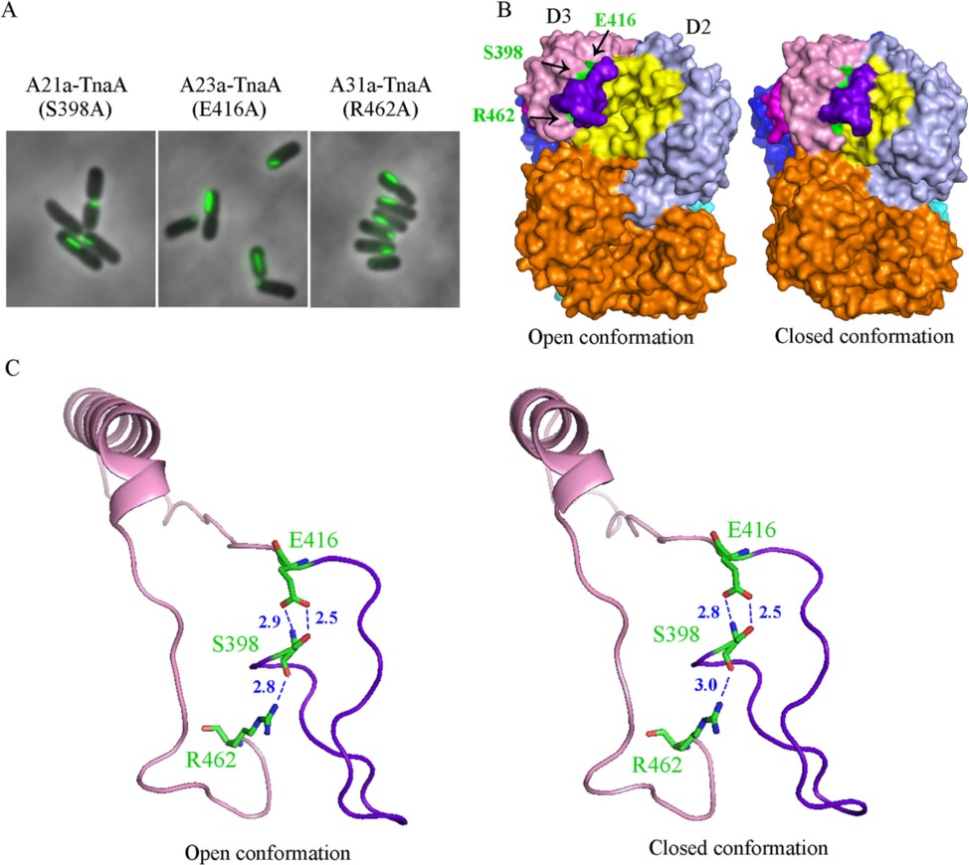
**Mutations that cause elongated ovoid focus assembly. A**. Single alanine substitution mutations for Ser398, Glu416 and Arg462 caused the TnaA-sfGFP variants to form elongated ovoid foci. The GFP images are overlaid with phase contrast images. **B**. The three residues (green) are mapped to the bottom of the loop (purple) at edge of catalytic pocket (yellow), on the crystal structures in the open (PDB 2OQX) and closed conformation (PDB 2C44) of TnaA. The structures are in side view compared to the structure in Figure 3A, with the same color codes. Only the upper catalytic pocket is highlighted. **C**. Ser398, Glu416 and Arg462 may form hydrogen bonds to stabilize the loop. Cartoon views of TnaA residues 398–462, with Ser398, Glu416 and Arg462 are illustrated in stick form. Nitrogen atoms (blue) and oxygen atoms (red) are marked. Possible hydrogen bonds between the three residues are illustrated, with distances indicated in Å.

The second mutant category consisted of TnaA variants that exhibited altered localization and/or enzymatic properties in cells as they reached the mid-to-late growth phase (Table 2). These mutants could be classified into three subgroups. The first subgroup consisted of full length proteins that exhibited delayed focus formation (Table 2, variants A2, A24 and A25). These variants were distributed diffusely in the cytoplasm during mid-log growth and formed a single polar or midcell focus about 30 min later than did wild type TnaA (e.g., Figure 6A). The mutated residues in these variants were located along the interface of neighboring D1D3-to-D1D3 domains (Figure 5A, deep blue residues), suggesting that interactions along the non-catalytic axis of the TnaA tetramer [32] might be affecting in vivo protein sequestration. The second subgroup, represented by only one mutant, localized normally when incorporated into the full length TnaA protein (Table 2, mutant A9, and Figure 6C). However, the corresponding A9-D1D3 truncation variant produced no foci but was, instead, distributed throughout the cytoplasm at all times (Table 2, and Figure 6D). The mutations in this variant were clustered tightly at the D1D3^1^-D2^3^ interface (Figure 5A, residues in cyan). However, the D2 domain was not present in this construct so that these residues were exposed on the surface of the A9-D1D3 truncation protein. Thus, these residues might be required for formation of dimers and foci. Finally, members of the third subgroup consisted of TnaA variants that produced highly elongated ovoid foci in the context of the full length mutant protein (Table 2, variants A21, A21a, A23, A23a, A31 and A31a). These ovoid foci (e.g., Figures 6E and 7A) were much different from the spherical foci associated with wild type TnaA and other variants (e.g., Figure 6C and F). These variants formed a single focus at midcell or at one pole, but the assemblies were elongated and oriented parallel to the long axis of the cell or, in some cases, were slightly tilted relative to the poles (Figures 6E and 7A). The mutations in these variant proteins clustered near the edge of the catalytic pocket, though they were not themselves part of the active site. A surprising observation was that some full-length TnaA variants localized differently than did their corresponding D1D3 truncation proteins (Table 2). For example, unlike their full length counterparts, focus formation was not delayed for the A2-D1D3, A24-D1D3 and A25-D1D3 truncation variants (Table 2 and Figure 6B). Also, as stated above, mutations at the D1D3^1^-D2^3^ interface produced a diffuse localization of A9-D1D3 (Figure 6D) but the A9-TnaA full-length variant continued to localize normally at the poles (Figure 6C). Finally, the mutations that caused full length TnaA-sfGFP to form elongated ovoid foci had no effect on the shape of foci produced by the truncation proteins A21-D1D3, A23-D1D3 or A31-D1D3 (Table 2, Figure 6F). These results suggest that polar localization is a complicated process with multiple interactions involving both the D1D3 and D2 domains.

The third major mutant category was composed of TnaA variants for which the disassembly of foci was delayed during stationary phase (Table 3). Previously, we showed that when wild type TnaA is expressed from its native promoter, the protein becomes more diffuse when entering stationary phase [25], an observation that was confirmed here because TnaA foci begin to disappear in early stationary phase cells (e.g., after 4–6 h, Table 4). In contrast, this third set of mutant proteins continued to form polar foci in almost 100% of cells at 6 h and began to become diffuse only at 12 h (Tables 3 and 4). Three of these variants also displayed altered focus formation (Table 2), suggesting that in some cases common factors may affect both assembly and disassembly of foci. In addition, A13-TnaA, which carries a Lys270Ala substitution at the active site, localized at poles even though it had no enzymatic activity (i.e., indole was produced at background levels) (Table 3, mutant A13). Other mutations in the catalytic pocket also eliminated indole production but had no effect on polar localization (e.g., A17-TnaA, Table 3). These results indicated that localization did not depend on the presence of the PLP cofactor or on the ability of TnaA to hydrolyze tryptophan. However, altering some active site residues did affect the disassembly of foci during stationary phase.

**Table 4:** **Indole activity of selected disassembly-delay mutants of TnaA**

**Variant** ^**a**^	**Cells with foci (%)**					**Indole production (mM)**				
	**2 h**	**3 h**	**4 h**	**6 h**	**12 h**	**2 h**	**3 h**	**4 h**	**6 h**	**12 h**
Wild type	100	100	100	75	67	0.02	0.25	0.55	0.59	0.50
A2	41	100	100	100	64	0.00	0.04	0.04	0.13	0.05
A9	100	100	100	100	72	0.01	0.05	0.03	0.12	0.07
A17	100	100	100	100	100	0.02	0.06	0.03	0.16	0.15
A21a	100	100	100	100	87	0.01	0.06	0.24	0.43	0.45

^a^The number of foci and enzyme activity of specified mutants of TnaA-sfGFP fusion proteins.

We particularly wished to know if delayed disassembly of TnaA foci was associated with a delay in the development of enzymatic activity, as measured by the production of indole. Therefore, we examined four of these variants (A2, A9, A17 and A21a) for enzymatic activity and for the presence of TnaA-sfGFP foci (Table 4). Each of the variants described in Tables 3 and 4 produced less indole at all points in the growth cycle, even though the amount of each variant protein was 2- to 3-fold higher per cell after 12 h incubation (not shown). In terms of TnaA localization, the selected proteins exhibited three types of behavior. First, the A21a variant most closely replicated the cycle of wild type TnaA-sfGFP. Although A21a foci disappeared later than did wild type TnaA foci, after 12 h the number of foci and enzymatic activity paralleled the results observed for wild type TnaA (Table 4, variant A21a). The A21a variant carries the S398A mutation, which is unable to close the peptide loop adjacent to the active site (Figure 7C). This implies that although the loop may inhibit substrate access to the active site (see Discussion), it may not be required for enzymatic activity per se. The A2 and A9 variants displayed a second type of behavior. For each of these proteins the number of foci decreased at 12 h, similar to wild type TnaA, but the enzymatic activity of the mutants remained low (Table 4). Therefore, these mutations may affect both localization and enzymatic activity. Finally, the A17 variant exhibited a third class of behavior – the number of cells with intact foci remained at almost 100%, even at 12 h, while enzymatic activity also remained low. This phenotype was consistent with the possibility that sequestered TnaA is inactive (see Discussion), though more work would have to be done to prove that this protein was active outside of foci.

### Three loop-associated substitutions produce elongated ovoid foci

**Figure 8:**
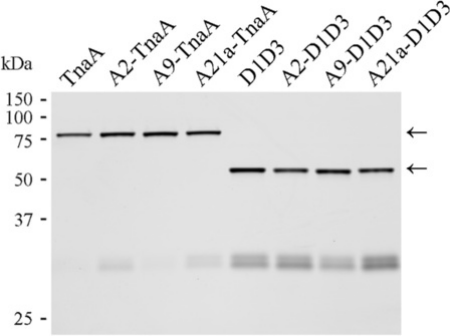
**Stability of TnaA-sfGFP and D1D3-sfGFP variants.** Cells expressing protein fusions that exhibited altered localization were grown in LB to mid-log phase (OD_600_ = 0.6) and analyzed by SDS-PAGE. Protein bands containing sfGFP were visualized by in-gel fluorescence imaging. The expected location of the full length fusion protein bands are denoted by arrows.

We were very intrigued by the formation of elongated ovoid foci in the A21-TnaA, A23-TnaA and A31-TnaA variants. Each of these proteins contained two to four amino acid substitutions, so we constructed single substitution variants to determine the residues responsible for this curious phenomenon. Each of three alanine substitutions replacing Ser398 (A21a-TnaA), Glu416 (A23a-TnaA) or Arg462 (A31a-TnaA) resulted in protein variants that formed elongated ovoid foci and decreased indole production by 43-78% (Table 2, Figure 7A). These altered localizations and enzyme activities were not due to decreased protein stability (Figure 8).

The three relevant residues mapped to the bottom of a loop, encompassing residues 398–416, located just to the side of the TnaA catalytic pocket (Figure 7B). This loop obscures the catalytic pocket in the closed conformation of TnaA (Figure 7B, right), but is canted away from the active site cavity in the open conformation of the protein (Figure 7B, left), making it likely that movement of this loop shifts the enzyme between its open-and-closed forms. The three residues are in close contact, possibly forming hydrogen bonds to maintain the position of this loop (Figure 7C). Surprisingly, the local arrangement of these residues and the loop itself in the closed form of TnaA are almost identical to the arrangements in the open form, suggesting that the movement of the loop is mediated mainly by moving the D1D3 domain towards the D2 domain. Mutations of the three residues may alter the position or orientation of the loop or may disrupt how the loop is tethered to the body of TnaA, thereby altering the protein’s enzymatic activity and localization. Interestingly, mutations that changed the composition of the loop itself (e.g., in variants A21b-TnaA, A21c-TnaA and A22-TnaA) did not affect polar localization or enzymatic activity (Table 1), suggesting that protein function depends more on loop placement than on loop composition. The results imply that the mutated residues at the base of the loop may alter the equilibrium between the open and closed conformations of TnaA by affecting loop dynamics.

## Discussion

A noteworthy number of proteins are distributed asymmetrically in bacterial cells, and these different locations may affect protein activity or function [33,34]. Such relationships are most obvious for proteins within macromolecular complexes, such as those that comprise the divisome, flagella, secretion systems and the chromosomal segregation apparatus [35-42]. It is less clear how or why other proteins are directed to specific locations, or whether differential localization affects protein function. However, a few examples lend credence to the idea that cellular localization causes or is at least associated with alterations in enzymatic activity. For example, the *E. coli* MurG protein participates in synthesizing peptidoglycan precursors for insertion into the cell wall, and excess amounts of this protein are localized to the cell poles, where the enzyme is apparently inactive [43]. Similarly, the activity of the *Bacillus subtilis* LicT antiterminator is accompanied by an overt change in its cellular localization: inactive enzyme (in the absence of the substrate salicin) is distributed diffusely in the cytoplasm, whereas active enzyme (in the presence of salicin) moves to a subpolar localization [44]. Finally, the *Caulobacter crescentus* CtpS protein, a CTP synthase, performs its enzymatic role when diffuse in the cytoplasm but also helps determine cell shape by polymerizing into a cytoskeletal filament along the cell membrane, this latter function being independent of its enzymatic activity [45]. We now report that the cellular location and activity of the TnaA tryptophanase are correlated, suggesting that the sequestration of TnaA in discrete foci may regulate its enzyme activity.

A strong indication that TnaA localization influences its enzymatic activity is that *E. coli* produces very little indole when TnaA is located in foci but that it produces large amounts of indole when the foci disappear and TnaA becomes diffuse throughout the cytoplasm. This suggests that the fraction of TnaA in foci is inactive and that the diffuse population is functional. A second argument supporting a relationship between protein function and focus formation is the existence of three mutations that reduce enzyme activity and change the geometry of TnaA foci from spherical to elongated ovals. The mechanism that creates these effects is unknown but the three altered residues are located at the base of a loop near the catalytic cavity, indicating that the disposition of this loop affects both characteristics. A third argument in support of the focus-function premise is that several mutations (i.e., in the mutants A2, A24 and A25) alter the kinetics or timing of focus formation and also reduce enzyme activity. The location of these mutated residues at the D1D3-to-D1D3 dimer interface, well away from the catalytic pocket, puts them in a position to affect the equilibrium among the different multimeric forms of TnaA, which may affect both sequestration and activity of the enzyme. Finally, several mutants delay the time when TnaA moves from foci to become diffuse while also delaying the time at which the enzyme becomes active. This strong genetic correlation suggests the two characteristics are causally related and corroborates the relationships observed for the wild type protein.

**Figure 9:**
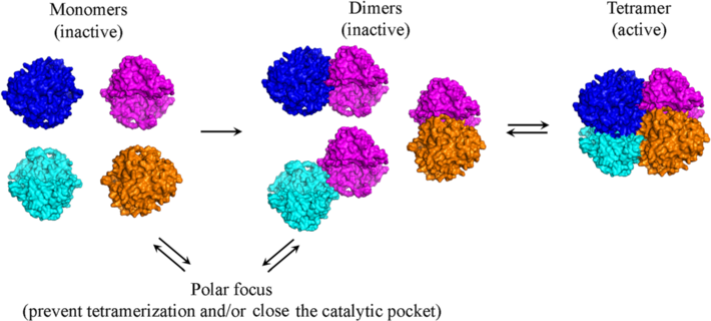
**Model of post-translational regulation of TnaA activity by focus formation.** Four TnaA units (magenta, blue, cyan and orange) were modeled as dimers and as the tetramer by using the PISA software, based on the crystal structure PDB 2OQX. Focus assembly of TnaA monomers and dimers would prevent the protein from assembling into active tetramers and/or trap the catalytic pocket in a closed conformation, thus regulating TnaA activity.

Overall, the results are consistent with a model in which individual foci contain inactive TnaA monomers or dimers while the diffuse fraction contains active tetramers (Figure 9). If true, then *E. coli* may regulate TnaA activity by altering the equilibrium between these protein states. This model is supported by previous work in which the TnaA C298S mutant, which is near the active site, disassociates more readily into inactive dimers, thus reinforcing the relationship between activity and tetramerization [32]. The fact that focus assembly is reversible, both *in vivo* during growth and *in vitro* when cells are disrupted, suggests that one or more cellular factors might help orchestrate the focus-to-diffuse shift. The physiological purpose of this regulation might be to restrict indole signaling to a particular time late in the growth cycle.

One of the most intriguing findings was that three individual missense mutations decrease the tryptophanase activity of TnaA and are associated with the formation of curiously elongated ovoid foci. The affected amino acids (Ser398, Glu416 and Arg462) are located at the base of a loop (from residues 398–416) that sits to one side of the catalytic pocket. In the two crystal structures of TnaA, this catalytic site is closed in one conformation and open in the other. The major cause of the closed pocket is a movement of the small domain (D1D3) towards the large domain (D2), re-orientating the loop so that it controls access to the active site by physically occluding the catalytic cavity. The three mutations that do change the behavior of TnaA foci are perfectly positioned to affect the orientation of the loop and may regulate the equilibrium between the open and closed versions of TnaA. Such alterations may also favor inter-molecular interactions that lead to the formation of oddly shaped foci. Alternately, the assembly of TnaA into foci may drive the loop to adopt one or the other orientation, which could determine whether the active site is more or less available to its substrate. Mutations affecting residues within the loop itself did not alter polar localization or enzymatic activity, implying that the loop’s orientation is the more important regulatory feature. However, combining any one of the three mutations at the base of the loop with mutations within the loop produced enzymes with even lower activity, suggesting that once the base is altered additional considerations come into play (Table 2, compare variants A21a, A23a and A31a to variants A21, A23 and A31). The tryptophanase of *Proteus vulgaris* and the tyrosine phenol-lyases of *Citrobacter freundii* and *Erwinia herbicola* are 51.7%, 41.1% and 43% identical to *E. coli* TnaA, respectively (EMBOSS Needle software analysis, via http://www.ebi.ac.uk/Tools/psa/emboss_needle/index.html), and all three have crystal structures with quaternary structures similar to that of *E. coli* TnaA (PDB entries 1AX4, 2EZ1 and 1C7G). Significantly, in all three enzymes the residues homologous to Ser398, Glu416 and Arg462 in *E. coli* TnaA are conserved and map to the base of a protruding loop near the active site, suggesting that the loop might play a similar structural and regulatory role in these enzymes, as well.

Lastly, we again address the question of whether polar assembly is an artifact caused by tagging TnaA with the fluorescent protein sfGFP. Mis-localization of analogous constructs made with the Clp protease [29] and MreB [46] does not mean that all protein fusion localizations are artifactual. The lesson is that such results need to be interpreted with caution when this kind of tagging is the sole approach for studying protein localization. In the case of TnaA, additional evidence suggests that polar assembly of TnaA-sfGFP is specific and likely represents its native localization [25]. Such evidence includes the fact that untagged TnaA associates with membrane vesicles derived from poles, that it competes with TnaA-sfGFP for polar localization, and that TnaA-sfGFP foci do not overlap with inclusion bodies [25]. Here, we find that the TnaA-sfGFP foci disappear when cells are lysed, again indicating that these are not inclusion bodies. Finally, when we fused TnaA to monomeric GFP (mGFPmut3) as recommended by Landgraf *et al.* [29], the composite protein formed the same type of polar foci, providing additional evidence that localization is not due to artifacts caused by sfGFP.

## Conclusions

The subcellular location and enzymatic activity of the TnaA tryptophanase are correlated, strongly suggesting that the sequestration of TnaA in discrete foci may regulate its enzyme activity. In addition, altering any one of three residues at the base of a loop near the catalytic pocket produces aberrant TnaA foci that exhibit greatly decreased tryptophanase activity. These latter results strongly imply that the disposition of this loop controls the formation of TnaA foci and may regulate the rate at which tryptophan can access the active site.

## Methods

### Strains, plasmids and growth conditions

All strains were derivatives of *E. coli* MG1655, and are listed in Table 5. Strain GL60 was the source for the *ibpA-mCherry::kan* allele [25]. The plasmid pTnaA (Kan^R^) carries the wild type *tnaA* gene under control of the *lac* promoter [25]. The plasmid pJDT1 (Amp^R^) contains *torA-gfpmut3* and was used as the template to amplify *gfpmut3* [47]. Strains were selected by adding kanamycin (Kan, 50 μg/ml) or ampicillin (Amp, 100 μg/ml). Cells were grown overnight in LB medium supplemented with 0.4% glucose. Overnight cultures were diluted 1:100 into 2 ml LB or M9 minimal medium supplemented with 2 mM MgSO_4_, 0.1 mM CaCl_2_, 33 μM thiamine, 1 % Bacto Casamino acids and 0.5 mM tryptophan. Cells were incubated with shaking at 37°C for TnaA localization and indole production experiments. When applicable, 50 μM IPTG was added to induce expression of the plasmid-encoded *tnaA* gene.

**Table 5:** ***E. coli*** **strains**

**Strain**	**Description** ^**a**^	**Source or Reference**
MG1655	Wild type F^−^ *λ* ^−^ *ilvG* ^−^ *rfb*-50 *rph*-1	Laboratory collection
GL38	MG1655 *aer-sfgfp::kan*	[25]
GL40	MG1655 *tnaA-sfgfp::kan*	[25]
GL60	MG1655 *ibpA-mCherry::kan*	[25]
GL504	MG1655 Δ*tnaA::frt*	[25]
GL619	GL607 Δ*tnaAB::frt* P_tufA_::*tnaA-sfgfp tnaB::frt*	[19]
GL676	MG1655 *tnaA-mgfpmut3::kan*	This study
GL678	MG1655 *D1-sfgfp::kan*	This study
GL680	MG1655 *D1D2-sfgfp::kan*	This study
GL682	GL504 *D3-sfgfp::kan*	This study
GL684	GL504 *D2D3-sfgfp::kan*	This study
GL686	GL504 *D1D3-sfgfp::kan*	This study
GL688	GL504 *D2-sfgfp::kan*	This study
GL726	GL686 *D1D3-sfgfp::frt*	This study
GL730	GL726 *D1D3-sfgfp::frt ibpA-mCherry::kan*	This study
GL734	GL504 *D1D3-mgfpmut3::kan*	This study
GL801	GL504 *A1-tnaA-sfgfp::kan* (K5A)	This study
GL802	GL504 *A2-tnaA-sfgfp::kan* (E9A, R12A, R14A)	This study
GL803	GL504 *A3-tnaA-sfgfp::kan* (E17A, K20A, R21A)	This study
GL804	GL504 *A4-tnaA-sfgfp::kan* (T23A, R24A, Y26A)	This study
GL805	GL504 *A5-tnaA-sfgfp::kan* (R27A, E28A, E29A)	This study
GL806	GL504 *A6-tnaA-sfgfp::kan* (K33A, S34A)	This study
GL807	GL504 *A7-tnaA-sfgfp::kan* (D42A, S43A, E44A, D45A)	This study
GL808	GL504 *A8-tnaA-sfgfp::kan* (D49A, T52A, D53A, S54A)	This study
GL809	GL504 *A9-tnaA-sfgfp::kan* (T60A, Q61A, S62A, Q64A)	This study
GL810	GL504 *A10-tnaA-sfgfp::kan* (K115A)	This study
GL811	GL504 *A11-tnaA-sfgfp::kan* (K156A)	This study
GL812	GL504 *A12-tnaA-sfgfp::kan* (K239A)	This study
GL813	GL504 *A13-tnaA-sfgfp::kan* (K270A)	This study
GL814	GL504 *A14-tnaA-sfgfp::kan* (N327A, D329A)	This study
GL815	GL504 *A15-tnaA-sfgfp::kan* (Q339A, Y340A, D343A)	This study
GL816	GL504 *A16-tnaA-sfgfp::kan* (E346A, E347A)	This study
GL817	GL504 *A17-tnaA-sfgfp::kan* (C352A, Q353A, Q354A)	This study
GL818	GL504 *A18-tnaA-sfgfp::kan* (D363A, K366A)	This study
GL819	GL504 *A19-tnaA-sfgfp::kan* (H370A, D374A, Q375A)	This study
GL820	GL504 *A20-tnaA-sfgfp::kan* (E384A, K387A, R392A)	This study
GL821	GL504 *A21-tnaA-sfgfp::kan* (S398A, R403A, D404A)	This study
GL822	GL504 *A22-tnaA-sfgfp::kan* (K406A, K409A, Q410A)	This study
GL823	GL504 *A23-tnaA-sfgfp::kan* (E416A, R419A)	This study
GL824	GL504 *A24-tnaA-sfgfp::kan* (T426A, Y427A, T428A)	This study
GL825	GL504 *A25-tnaA-sfgfp::kan* (Q429A, T430A, H431A, D433A)	This study
GL826	GL504 *A26-tnaA-sfgfp::kan* (E437A, K440A, H441A)	This study
GL827	GL504 *A27-tnaA-sfgfp::kan* (K443A, E444A, N445A, N448A)	This study
GL828	GL504 *A28-tnaA-sfgfp::kan* (K450A)	This study
GL829	GL504 *A29-tnaA-sfgfp::kan* (T453A, T455A, Y456A, E457A)	This study
GL830	GL504 *A30-tnaA-sfgfp::kan* (K459A)	This study
GL831	GL504 *A31-tnaA-sfgfp::kan* (R462A, H463A, T465A)	This study
GL832	MG1655 *A32-tnaA-sfgfp::kan* (K467A, K469A, E470A)	This study
GL833	GL504 *A21a-tnaA-sfgfp::kan* (S398A)	This study
GL834	GL504 *A21b-tnaA-sfgfp::kan* (R403A)	This study
GL835	GL504 *A21c-tnaA-sfgfp::kan* (D404A)	This study
GL836	GL504 *A23a-tnaA-sfgfp::kan* (E416A)	This study
GL837	GL504 *A23b-tnaA-sfgfp::kan* (R419A)	This study
GL838	GL504 *A31a-tnaA-sfgfp::kan* (R462A)	This study
GL839	GL504 *A31b-tnaA-sfgfp::kan* (H463A)	This study
GL840	GL504 *A31c-tnaA-sfgfp::kan* (T465A)	This study
GL844	GL504 *A33-tnaA-sfgfp::kan* (K5A, K115A, K156A, K239A, K450A, K459A)	This study
GL802D	GL504 *A2-D1D3-sfgfp::kan* (E9A, R12A, R14A)	This study
GL803D	GL504 *A3-D1D3-sfgfp::kan* (E17A, K20A, R21A)	This study
GL804D	GL504 *A4-D1D3-sfgfp::kan* (T23A, R24A, Y26A)	This study
GL805D	GL504 *A5-D1D3-sfgfp::kan* (R27A, E28A, E29A)	This study
GL806D	GL504 *A6-D1D3-sfgfp::kan* (K33A, S34A)	This study
GL807D	GL504 *A7-D1D3-sfgfp::kan* (D42A, S43A, E44A, D45A)	This study
GL808D	GL504 *A8-D1D3-sfgfp::kan* (D49A, T52A, D53A, S54A)	This study
GL809D	GL504 *A9-D1D3-sfgfp::kan* (T60A, Q61A, S62A, Q64A)	This study
GL814D	GL504 *A14-D1D3-sfgfp::kan* (N327A, D329A)	This study
GL815D	GL504 *A15-D1D3-sfgfp::kan* (Q339A, Y340A, D343A)	This study
GL816D	GL504 *A16-D1D3-sfgfp::kan* (E346A, E347A)	This study
GL817D	GL504 *A17-D1D3-sfgfp::kan* (C352A, Q353A, Q354A)	This study
GL818D	GL504 *A18-D1D3-sfgfp::kan* (D363A, K366A)	This study
GL819D	GL504 *A19-D1D3-sfgfp::kan* (H370A, D374A, Q375A)	This study
GL820D	GL504 *A20-D1D3-sfgfp::kan* (E384A, K387A, R392A)	This study
GL821D	GL504 *A21-D1D3-sfgfp::kan* (S398A, R403A, D404A)	This study
GL822D	GL504 *A22-D1D3-sfgfp::kan* (K406A, K409A, Q410A)	This study
GL823D	GL504 *A23-D1D3-sfgfp::kan* (E416A, R419A)	This study
GL824D	GL504 *A24-D1D3-sfgfp::kan* (T426A, Y427A, T428A)	This study
GL825D	GL504 *A25-D1D3-sfgfp::kan* (Q429A, T430A, H431A, D433A)	This study
GL826D	GL504 *A26-D1D3-sfgfp::kan* (E437A, K440A, H441A)	This study
GL827D	GL504 *A27-D1D3-sfgfp::kan* (K443A, E444A, N445A, N448A)	This study
GL829D	GL504 *A29-D1D3-sfgfp::kan* (T453A, T455A, Y456A, E457A)	This study
GL831D	GL504 *A31-D1D3-sfgfp::kan* (R462A, H463A, T465A)	This study
GL832D	GL504 *A32-D1D3-sfgfp::kan* (K467A, K469A, E470A)	This study
GL833D	GL504 *A21a-D1D3-sfgfp::kan* (S398A)	This study

^a^ *frt* indicates the presence of the following oligonucleotide scar that was left after removing the *kan* cassette: GAAGTTCCTATACTTTCTAGAGAATAGGAACTTC. The “*A##*” numbers correspond to the site-directed mutations described in Tables 1 and 2.

### Strain construction

Chromosomal gene deletions or insertions were performed by using λ-Red recombination [48]. The DNA fragments were amplified by one-step or sequential PCR using designated primers (see Additional file 1). To construct the D1-sfGFP and D1D2-sfGFP strains, the *sfgfp::kan* cassettes were amplified from the chromosome of *E. coli* GL38 (*aer-sfgfp::kan*) and inserted into the chromosome of MG1655 to delete the appropriate subdomain fragments and to fuse *sfgfp* to *D1* and *D1D2*. To construct the D2-sfGFP, D3-sfGFP, D2D3-sfGFP and D1D3-sfGFP strains, the respective *sfgfp*-containing fragments were amplified from the chromosome of GL680 (*D1D2-sfgfp::kan*) or GL40 (*tnaA-sfgfp::kan*) and inserted into the chromosome of GL504 (Δ*tnaA::frt*) under control of the original *tna* promoter. The A1-TnaA-sfGFP to A31-TnaA-sfGFP strains were constructed by amplifying the *tnaA-sfgfp::kan* fragments from the chromosome of GL40 and inserting them into the chromosome of GL504 at the original *tnaA* gene location. To construct the A32-TnaA-sfGFP strain, the *sfgfp::kan* fragment was amplified from the chromosome of *E. coli* GL38 and inserted into the chromosome of MG1655 to introduce the mutation and to fuse *sfgfp* to *tnaA*. To construct the A33-TnaA-sfGFP strain, the DNA fragment was generated by six rounds of PCR reactions to introduce the mutations for the six codons one by one. The A1-D1D3-sfGFP to A32-D1D3-sfGFP fusions were constructed in the same way as described for the D1D3-sfGFP fusions, except that the chromosomes of corresponding A1-TnaA-sfGFP to A32-TnaA-sfGFP strains were used as the templates for PCR. To construct the TnaA-mGFPmut3 fusion, the fragment encoding mGFPmut3 was amplified from *gfpmut3* in pJDT1 by using two-step PCR to introduce the A206K mutation [29]. The *mgfpmut3* fragment was joined to the *kan* cassette by an additional PCR and then inserted into the chromosome of MG1655 to replace the stop codon of *tnaA*. The D1D3-mGFPmut3 fusion was constructed as described for the D1D3-sfGFP fusion, except that the relevant gene fragment was amplified from the chromosome of GL676 (*tnaA-mgfpmut3::kan*). The entire *tnaA* region in all strains was PCR amplified and sequenced to confirm the presence of the correct gene constructions.

### TnaA structure modeling

The crystal structures of TnaA (open conformation, PDB 2OQX; closed conformation, PDB 2C44) were assembled into tetramers by using the PISA software (http://www.ebi.ac.uk/pdbe/prot_int/pistart.html) [49]. *In silico* analysis and visualization of TnaA structures were performed by using the PyMOL Molecular Graphics System, Version 1.3. Schrödinger, LLC (http://www.pymol.org).

### Microscopy

Phase contrast and fluorescence images of cells were obtained by using an Olympus microscope BX60 with a 100× oil objective (1.3 NA PH3), a 1.4 MP MONO CCD camera XM10, and illuminated by the fluorescence source X-Cite120. Live cells were loaded to 1% agarose-coated slides for imaging. GFP was visualized by using the OSF-0008Z filter set (471 nm excitation/520 nm emission).

### TnaA enzyme activity and protein stability

TnaA activity was assayed by measuring indole production [19]. Standard curves with known concentrations of indole confirmed that the assay was linear for indole amounts down to 0.01 mM. Samples were collected at different points during cell growth and the amounts of indole were determined as described previously [19]. Cellular proteins were separated by SDS-PAGE, and TnaA and its various protein fragments that had been fused to sfGFP were visualized by in-gel GFP fluorescence imaging [19].

## Additional file

Additional file 1: Table S1. Title of dataset: Oligonucleotides used to construct *tnaA* mutants. Description of dataset: list of oligonucleotides used in making mutant genes.
