# Supplementary material for: A new suite of tnaA mutants suggests that Escherichia coli tryptophanase is regulated by intracellular sequestration and by occlusion of its active site
Source: BMC Microbiol. 2015 Feb 4;15(1):14. doi: 10.1186/s12866-015-0346-3 (PMC4323232; doi:10.1186/s12866-015-0346-3)
Supplement: Additional file 1: Table S1. — Title of dataset: Oligonucleotides used to construct tnaA mutants. Description of dataset: list of oligonucleotides used in making mutant genes. [file 12866_2015_346_MOESM1_ESM.pdf]

Additional Table S1. Oligonucleotides used to construct *tnaA* mutants.

Description	Oligonucleotides (5’-3’)*
tnaA-up primer	*gtaatattcacagggatcactg*
tnaA-down primer	*agtggctaacatccttatagccactctgtagtattaatta*tgaatatcctccttagttcc
D1-sfgfp::kan	*gaccgacagcggcaccggggcggtgacgcagagcatgcag*gcaggtgactacaaagacga F1<br>*tnaA-down* R1
D1D2-sfgfp::kan	*aggcggcgcgatggagcgtctggcggtaggtctgtatgac*gcaggtgactacaaagacga F1<br>*tnaA-down* R1
D3-sfgfp::kan	*gggatcactgtaattaaaataaatgaaggattatgtaatg*ggcatgaatctcgactggct F1<br>*tnaA-down* R1
D2D3-sfgfp::kan	gggatcactgtaattaaaataaatgaaggattatgtaatggctgcgatgatgcgcggcga F1<br>*tnaA-down* R1
D1D3-sfgfp::kan	*tnaA-up* F1<br>agcctgcatgctctgcgtca R1<br>tgacgcagagcatgcaggctgcgggttatgacggcatgaatctcgactgg F2<br>*tnaA-down* R2
D2-sfgfp::kan	gggatcactgtaattaaaataaatgaaggattatgtaatggctgcgatgatgcgcggcga F1<br>*tnaA-down* R1
A1-tnaA-sfgfp::kan	*gggatcactgtaattaaaataaatgaaggattatgtaatg*gaaaactttGCacatctccctgaa<br>ccgttcc F1<br>*tnaA-down* R1
A2-tnaA-sfgfp::kan	*tnaA-up* F1<br>agggagatgtttaaagttttcc R1<br>ggaaaactttaaacatctccctgCaccgttcGCcattGCtgttattgagccagtaaaacg F2<br>*tnaA-down* R2
A3-tnaA-sfgfp::kan	*tnaA-up* F1<br>aataacacgaatgcggaacgg R1<br>ccgttccgcattcgtgttattGCgccagtaGCaGCtaccactcgcgcttatcgtg F2<br>*tnaA-down* R2
A4-tnaA-sfgfp::kan	*tnaA-up* F1<br>ggtacgttttactggctcaataac R1<br>gttattgagccagtaaaacgtaccGctGCcgctGCtcgtgaagaggcaattattaaatcc F2<br>*tnaA-down* R2
A5-tnaA-sfgfp::kan	*tnaA-up* F1<br>ataagcgcgagtggtacgttttac R1<br>gtaaaacgtaccactcgcgcttatGCtgCagCggcaattattaaatccggtatg F2<br>*tnaA-down* R2
A6-tnaA-sfgfp::kan	*tnaA-up* F1<br>aataattgcctcttcacgataagc R1<br>gcttatcgtgaagaggcaattattGCaGccggtatgaacccgttcctgctg F2<br>*tnaA-down* R2
A7-tnaA-sfgfp::kan	*tnaA-up* F1<br>cagcaggaacgggttcatacc R1<br>ggtatgaacccgttcctgctggCtGCcgCagCtgtttttatcgatttactgacc F2<br>*tnaA-down* R2
A8-tnaA-sfgfp::kan	*tnaA-up* F1<br>gataaaaacatcttcgctatcc R1<br>ggatagcgaagatgtttttatcgCtttactgGccgCTGCcggcaccggggcggtgacgca F2<br>*tnaA-down* R2

Description	Oligonucleotides (5’-3’)*
A9-tnaA-sfgfp::kan	*tnaA-up* F1<br>caccgccccggtgccgctgtcgg R1<br>ccgacagcggcaccggggcggtgGcgGCAGCcatgGCggctgcgatgatgcgcggcgac F2<br>*tnaA-down* R2
A10-tnaA-sfgfp::kan	*tnaA-up* F1<br>aatcagtaccggaatatagatttg R1<br>caaatctatattccggtactgattGCaaaacgcgagcaggaaaaagg F2<br>*tnaA-down* R2
A11-tnaA-sfgfp::kan	*tnaA-up* F1<br>gatatagacgttacgcacgg R1<br>ccgtgcgtaacgtctatatcGCagaagccttcgatacgggc F2<br>*tnaA-down* R2
A12-tnaA-sfgfp::kan	*tnaA-up* F1<br>gatgaaataggcgttttcag R1<br>ctgaaaacgcctatttcatcGCAcagcgtgaagcagaatacaaag F2<br>*tnaA-down* R2
A13-tnaA-sfgfp::kan	*tnaA-up* F1<br>ggcggacatcgccagcatatc R1<br>gatatgctggcgatgtccgccaagGCagatgcgatggtgccgatgg F2<br>*tnaA-down* R2
A14-tnaA-sfgfp::kan	*tnaA-up* F1<br>catgccgtcatacagacctacc R1<br>gcggtaggtctgtatgacggcatgGCtctcgCctggctggcttatcgtatcgc F2<br>*tnaA-down* R2
A15-tnaA-sfgfp::kan	*tnaA-up* F1<br>tacctgcgcgatacgataagcc R1<br>ctggcttatcgtatcgcgcaggtaGCgGCtctggtcgCtggtctggaagagattggcg F2<br>*tnaA-down* R2
A16-tnaA-sfgfp::kan	*tnaA-up* F1<br>cagaccatcgaccagatactgtac R1<br>gtacagtatctggtcgatggtctggCagCgattggcgttgtctgccagcaggcgggcggtcacg<br>cggcattcgttgatgc F2<br>*tnaA-down* R2
A17-tnaA-sfgfp::kan	*tnaA-up* F1<br>gacaacgccaatctcttccagac R1<br>ggtctggaagagattggcgttgtcGCcGCTGCAgcgggcggtcacgcggcattc F2<br>*tnaA-down* R2
A18-tnaA-sfgfp::kan	*tnaA-up* F1<br>caacgaatgccgcgtgacc R1<br>caggcgggcggtcacgcggcattcgttgCtgccggtGCactgttgccgcatatcccgg F2<br>*tnaA-down* R2
A19-tnaA-sfgfp::kan	*tnaA-up* F1<br>cggcaacagtttaccggcatc R1<br>ttgatgccggtaaactgttgccgGCtatcccggcagCcGCgttcccggcacaggcgct F2<br>*tnaA-down* R2
A20-tnaA-sfgfp::kan	*tnaA-up* F1<br>cgcaggccagcgcctgtg R1<br>gttcccggcacaggcgctggcctgcgCgctgtatGCagtcgccggtatcGCtgcggtagaaatt<br>ggctctttc F2<br>*tnaA-down* R2
A21-tnaA-sfgfp::kan	*tnaA-up* F1<br>gccaatttctaccgcacggatac R1<br>gtatccgtgcggtagaaattggcGctttcctgttaggcGCcgCtccgaaaccggtaaac F2<br>*tnaA-down* R2

Description	Oligonucleotides (5’-3’)*
A22-tnaA-sfgfp::kan	*tnaA-up* F1<br>cggatcgcggcctaacag R1<br>gctctttcctgttaggccgcgatccgGCaaccggtGCaGCactgccatgcccggctg F2<br>*tnaA-down* R2
A23-tnaA-sfgfp::kan	*tnaA-up* F1<br>cagccgggcatggcag R1<br>ccggtaaacaactgccatgcccggctgCactgctgGCtttaaccattccgcgcgcaac F2<br>*tnaA-down* R2
A24-tnaA-sfgfp::kan	*tnaA-up* F1<br>tgcgcgcggaatggttaaacg R1<br>tgctgcgtttaaccattccgcgcgcaGCaGCtGctcaaacacatatggacttcattattg F2<br>*tnaA-down* R2
A25-tnaA-sfgfp::kan	*tnaA-up* F1<br>agtatatgttgcgcgcggaatg R1<br>ccattccgcgcgcaacatatactGCaGcaGCtatggCcttcattattgaagcctttaaac F2<br>*tnaA-down* R2
A26-tnaA-sfgfp::kan	*tnaA-up* F1<br>caataatgaagtccatatgtgtttgag R1<br>ctcaaacacatatggacttcattattgCagcctttGCaGCtgtgaaagagaacgcggcgaatat<br>taaaggattaacc F2<br>*tnaA-down* R2
A27-tnaA-sfgfp::kan	*tnaA-up* F1<br>cacatgtttaaaggcttcaataatgaag R1<br>cttcattattgaagcctttaaacatgtgGCagCgGCcgcggcgGCtattaaaggattaacccttt<br>acgtacgaacc F2<br>*tnaA-down* R2
A28-tnaA-sfgfp::kan	*tnaA-up* F1<br>aatattcgccgcgttctc R1<br>gagaacgcggcgaatattGCaggattaacctttacgtacg F2<br>*tnaA-down* R2
A29-tnaA-sfgfp::kan	*tnaA-up* F1<br>taatcctttaatattcgccgcgttc R1<br>gagaacgcggcgaatattaaaggattaGcctttGCgGCTgCaccgaaagtattgcgtcac F2<br>*tnaA-down* R2
A30-tnaA-sfgfp::kan	*tnaA-up* F1<br>cggttcgtacgtaaaggttaatc R1<br>gattaacctttacgtacgaaccgGCagtattgcgtcacttcaccg F2<br>*tnaA-down* R2
A31-tnaA-sfgfp::kan	*tnaA-up* F1<br>caatactttcggttcgtacgtaaagg R1<br>aacctttacgtacgaaccgaaagtattgGCtGCcttcGccgcaaaacttaaagaagttgc F2<br>*tnaA-down* R2
A32-tnaA-sfgfp::kan	*ttaacctttacgtacgaaccgaaagtattgcgtcacttcaccgcaGCacttGCagCagtt*gcag<br>gtgactacaaagacga F1<br>*tnaA-down* R1
A21a-tnaA-sfgfp::kan	*tnaA-up* F1<br>gccaatttctaccgcacggatac R1<br>gtatccgtgcggtagaaattggcGctttcctgttaggccgcgatccgaaaaccggtaaac F2<br>*tnaA-down* R2
A21b-tnaA-sfgfp::kan	*tnaA-up* F1<br>gccaatttctaccgcacggatac R1<br>gtatccgtgcggtagaaattggctctttcctgttaggcGCcgatccgaaaaccggtaaac F2<br>*tnaA-down* R2

Description	Oligonucleotides (5’-3’)*
A21c-tnaA-sfgfp::kan	*tnaA-up* F1<br>gccaatttctaccgcacggatac R1<br>gtatccgtgcggtagaaattggctctttcctgttaggccgcgCtccgaaaaccggtaaac F2<br>*tnaA-down* R2
A23a-tnaA-sfgfp::kan	*tnaA-up* F1<br>cagccgggcatggcag R1<br>ccggtaaacaactgccatgcccggctgCactgctgcgtttaaccattccg F2<br>*tnaA-down* R2
A23b-tnaA-sfgfp::kan	*tnaA-up* F1<br>cagccgggcatggcag R1<br>ccggtaaacaactgccatgcccggctgaactgctgGCtttaaccattccgcgcgcaac F2<br>*tnaA-down* R2
A31a-tnaA-sfgfp::kan	*tnaA-up* F1<br>caatactttcggttcgtacgtaaagg R1<br>aacctttacgtacgaaccgaaagtattgGCtcacttcaccgcaaaacttaaagaagttgc F2<br>*tnaA-down* R2
A31b-tnaA-sfgfp::kan	*tnaA-up* F1<br>caatactttcggttcgtacgtaaagg R1<br>aacctttacgtacgaaccgaaagtattgcgtGCcttcaccgcaaaacttaaagaagttgc F2<br>*tnaA-down* R2
A31c-tnaA-sfgfp::kan	*tnaA-up* F1<br>caatactttcggttcgtacgtaaagg R1<br>aacctttacgtacgaaccgaaagtattgcgtcacttcGccgcaaaacttaaagaagttgc F2<br>*tnaA-down* R2
D1D3-sfgfp::kan substitution mutants	Same as *D1D3-sfgfp::kan*, except for *A9-D1D3-sfgfp::kan* and *A14-D1D3-sfgfp::kan*
A9-D1D3-sfgfp::kan	*tnaA-up* F1<br>agccGCcatgGCTGCcgCca R1<br>tgGcgGCAGCcatgGCggctgcgggttatgacggcatgaatctcgactg F2<br>*tnaA-down* R2
A14-D1D3-sfgfp::kan	*tnaA-up* F1<br>agcctgcatgctctgcgtca R1<br>tgacgcagagcatgcaggctgcgggttatgacggcatgGCtctcgCctg F2<br>*tnaA-down* R2
tnaA-mgfpmut3::kan	*caggtgactacaaagacgatgacgacaag*ggtctgtctaaaggagaagaacttttcactg F1<br>agattgtgtggacaggtaatg R1<br>cattacctgtccacacaatctAAGctttcgaaagatcccaacg F2<br>cagccaattgaccggtcctattatttgtatagttcatccatgcc R2<br>taggaccggtcaattggctg F3<br>*tnaA-down* R3
D1D3-mgfpmut3::kan	*Same as D1D3-sfgfp::kan*

* For sequential PCR, the first fragment was generated by using primers F1 and R1, the second fragment was generated by using F2 and R2, and the fragments were connected by a third PCR using F1 and R2. For construction of *tnaA-mgfpmut3::kan*, the first two fragments were connected to encode *mgfpmut3*, and the third fragment was the *kan* cassette. See strain construction for the DNA templates used in PCR. Italic type in oligonucleotides: sequences homologous to the target chromosomal sequence. Uppercase: altered nucleotides that create substitution mutations.
